# The Transmission Route and Selection Pressure in HCV Subtype 3a and 3b Chinese Infections: Evolutionary Kinetics and Selective Force Analysis

**DOI:** 10.3390/v14071514

**Published:** 2022-07-11

**Authors:** Ru Xu, Xia Rong, Elihu Aranday-Cortes, Sreenu Vattipally, Joseph Hughes, John McLauchlan, Yongshui Fu

**Affiliations:** 1 Institute of Clinical Blood Transfusion, Guangzhou Blood Center, Guangzhou 510095, China; leng868@163.com (R.X.); joyjoy@126.com (X.R.); 2 The Key Medical Laboratory of Guangzhou, Guangzhou 510095, China; 3 School of Laboratory Medicine and Biotechnology, Southern Medical University, Guangzhou 510515, China; 4 Garscube Campus, MRC-University of Glasgow Centre for Virus Research, Glasgow G61 1QH, UK; elihu.aranday-cortes@glasgow.ac.uk (E.A.-C.); sreenu.vattipally@glasgow.ac.uk (S.V.); joseph.hughes@glasgow.ac.uk (J.H.)

**Keywords:** hepatitis C virus, evolution, selection, transfusion route, origin

## Abstract

Hepatitis C virus (HCV) genotype 3 (GT-3) represents 22–30% of all infections and is the second most common genotype among all HCV genotypes. It has two main subtypes, GT-3a and GT-3b, that present epidemiological differences in transmission groups. This report generated 56 GT-3a and 64 GT-3b whole-genome sequences to conduct an evolutionary kinetics and selective force analysis with reference sequences from various countries. Evolutionary analysis showed that HCV GT-3a worldwide might have been transmitted from the Indian subcontinent to South Asia, Europe, North America and then become endemic in China. In China, GT-3a may have been transmitted by intravenous drug users (IDUs) and become endemic in the general population, while GT-3b may have originated from IDUs and then underwent mutual transmission between blood donors (BDs) and IDUs, ultimately becoming independently endemic in IDUs. Furthermore, the spread of GT-3a and GT-3b sequences from BD and IDU populations exhibit different selective pressures: the proportion of positively selected sites (PPSs) in E1 and E2 from IDUs was higher than in BDs. The number of positive selection sites was higher in GT-3b and IDUs. These results indicate that different selective constraints act along with the GT-3a and GT-3b genomes from IDUs and BDs. In addition, GT-3a and GT-3b have different transmission routes in China, which allows us to formulate specific HCV prevention and control strategies in China.

## 1. Introduction

Hepatitis C virus (HCV) is a major cause of severe liver disease, including chronic hepatitis, liver cirrhosis, and cancer. The prevalence of anti-HCV antibodies in China is approximately 0.91%, representing 10 million HCV infections that account for more than 14% of the global population infected with HCV [1]. HCV is phylogenetically divided into eight genotypes (gt1-8) and 90 subtypes (https://talk.ictvonline.org/ictv_wikis/flaviviridae/w/sg_flavi/634/table-1 accessed on 1 February 2022). Genotype 3 (GT-3) represents 22–30% of all HCV infections and is the second most common genotype among all HCV genotypes [2]. GT-3 infection is associated with higher rates of steatosis, accelerated development of cirrhosis, and higher risk of developing hepatocellular carcinoma (HCC) [3]. Although direct-acting antivirals (DAA) for HCV treatment have greatly lessened the burden of HCV, soluble inflammatory signatures remain high and are linked to hepatic damage even after DAA-mediated clearance of HCV [4,5]. Advanced cirrhosis or prior treatment experience combined with the presence of resistance-associated substitutions (RASs) may result in GT-3 DAA treatment failure [6]. The prevalence of GT-3 infection is high in intravenous drug users (IDUs), and the proportion has increased in recent years [7,8]. Rising numbers of IDUs increase the risk of HCV transmission from this high-risk population to the general population, heightening the possibility of more GT-3 infection.

The most common subtypes of GT-3 are GT-3a and GT-3b. These two subtypes display epidemiological differences that influence their phylodynamics. GT-3a is the most prevalent GT-3 subtype in North America (98.7%), Europe (98.9%), and Oceania (98.7%) [9]. In India, about 56% of patients are infected with GT-3a. Subtype GT-3b (20.3%) is also present in the Indian population and is rarely found outside Asia [9]. Although GT-3a is widely distributed, there is only a relatively small percentage of GT-3a infections in China (1.7–11.38%) [10,11,12]. GT-3b, which represents more than 50% of GT-3 infections in China, is endemic in southwest and south China [10]. In Sichuan, Yunnan, Chongqing, Guizhou, and Guangdong provinces, GT-3b was the second predominant subtype, accounting for 8.47%, 20.52%, 21.86%, 21.93%, and 20.9% of infections among all genotypes, respectively [11,13]. GT-3a originally emerged and diversified in Southeast Asia and the Indian subcontinent [14], while a common origin of GT-3b was found in Thailand according to the analysis of sequence data from the HCV E1 region [12]. Clinical differences may also exist: the sustained viral response (SVR) after 12 weeks of treatment with sofosbuvir plus ribavirin has been reported to be significantly lower for subtype GT-3b compared with GT-3a [15]. There are no reports on the differences between these two subtypes at the genetic level because few GT-3b full-length genomes have been archived in public databases.

Due to the lack of any proof-reading activity in RNA-dependent RNA polymerase (NS5B), and the pressure exerted by the host immune response, HCV displays high genetic variability. Like most RNA viruses, HCV evolves rapidly, and mutations are the major cause of genetic variation as recombination between HCV genomes is rare. Transmission of the virus has been reported to be an instrumental force in driving HCV mutations and evolution [16]. When the virus is transmitted to a new host, immune pressure allows the virus to accumulate non-synonymous mutations, enabling escape from the immune system along with gains in fitness advantage as infection of the individual progresses to chronicity [17]. Our goal in this study was to compare the characteristics and extent of genetic variation, evolutionary kinetics, and selective forces driving the evolution of HCV sequences between the GT-3a and GT-3b subtypes from IDU and blood donor (BD) populations. First, we determined the entire HCV GT-3a and GT-3b genome sequences from IDU and BD populations. Then, a comprehensive evolutionary analysis was performed to reveal the degree of HCV mutation and propagation mode in IDUs and BDs in China. We found that the proportion of positively selected sites (PPSs) in E1 and E2 from IDUs was higher than in BDs, while the number of positive selection sites was higher in GT-3b and IDUs. This information will aid improvements in HCV prevention and control strategies, reducing the risk of exposure to the virus in health care settings and high-risk populations.

## 2. Materials and Methods

### 2.1. Sample Collection

A BD cohort of 493 HCV infections was recruited from Guangzhou blood donors, and an IDU cohort was from our previous study [11]. The HCV genotype was determined by sequence analysis of the NS5B and E1 regions [11,18]. The institutional review board approved this study at the Guangzhou Blood Center, and the guidelines set by this board were strictly followed. The study protocols followed the ethical guidelines set in place by the 1975 Declaration of Helsinki and were approved by the Medical Ethics Committee of Guangzhou Blood Center. All samples included in this analysis are baseline samples collected from treatment-naïve HCV-positive individuals.

### 2.2. Next-Generation Sequencing and Bioinformatic Processing

Total RNA was extracted from 200 μL plasma using the AgencourtRNAdvance blood kit (Beckman Coulter, CA, USA) and then reverse-transcribed using Superscript III (Invitrogen, CA, USA). The preparation of libraries and the sequencing were performed as in a previous study [19]. Briefly, double-stranded DNA was generated with an NEB Second Strand Synthesis kit (New England BioLabs, Ipswich, MA, USA). After that, the KAPA Library Prep kit (KAPA Biosystems, Boston, MA, USA) was used to prepare libraries and pooled at equimolar concentrations for sequencing on the Illumina MiSeq platform (v3 chemistry). Bioinformatic processing was performed as before [19]. First, the raw reads were trimmed using trim_galore (https://www.bioinformatics.babraham.ac.uk/projects/trim_galore/ accessed on 3 July 2022). Next, the clean reads were enriched in silico by removing the human and rRNA reads (Ribopicker, http://ribopicker.sourceforge.net/, accessed on 3 July 2022) and then matched against whole-genome HCV reference sequences using Tanoti (http://bioinformatics.cvr.ac.uk/tanoti.php, accessed on 3 July 2022). For GT-3b, because there are only 14 full-length sequences in NCBI, these reads were iteratively assembled using the SPAdes de novo assembler [20]. Finally, the same files were generated using Tanoti, and a majority consensus sequence was generated for sites with >5 reads.

### 2.3. HCV Genotyping and Sequence Datasets

The consensus sequences were placed within the context of previously known genotypes (https://talk.ictvonline.org/ictv_wikis/flaviviridae/w/sg_flavi/56/hcv-classification) (accessed on 1 February 2022) using the MEGA X software package and the maximum likelihood method (using the general time-reversible model and gamma distributed with invariant site (G + I) rate) with 500 bootstrap replicates with partial deletion for missing data. RDP4 and GARD were used to test for recombination. All available HCV GT-3 full-length reference sequences were downloaded from NCBI (https://www.ncbi.nlm.nih.gov/gene, accessed on 3 July 2022) and the HCV-GLUE website (http://hcv-glue.cvr.ac.uk/, accessed on 3 July 2022) [21]. The available HCV full-length sequence dataset contained similar representations for each subtype except for GT-3a and 3b, which contained 580 and 14 reference sequences, respectively. Thirty-seven representative GT-3a and eight 3b reference sequences were selected according to the topology of the phylogenetic tree taking account of the location and the sample dates of the reference sequences to avoid an over-representation of subtypes GT-3a and 3b when calculating the intra-GT-3 distances. Consequently, a total of 179 GT-3 sequences were aligned using MAFFT [22,23] and used for all subsequent analyses (detailed reference sequences are shown in Supplementary Table S1). The sequence set that was further analyzed below contained GT-3a subsets (37 references and 56 Chinese sequences), GT-3b subsets (eight references and 64 Chinese sequences), Chinese IDU subsets (15 GT-3a IDUs and 43 GT-3b IDUs), and Chinese BD subsets (41 GT-3a BDs and 21 GT-3b BDs).

### 2.4. Entropy and Diversity Measurement

The sequence distance within SSE Alignment software (http://www.virus-evolution.org/Downloads/Software/, accessed on 3 July 2022) was estimated to present the graphical results of the distance comparisons between GT-3a and GT-3b subsets. Shannon Entropy-two analysis (https://www.hiv.lanl.gov/content/sequence/ENTROPY/entropy.html, accessed on 3 July 2022) was used on the Los Alamos web server using 100 randomizations to evaluate the sequence diversity from GT-3a and GT-3b subsets. Entropy values for each amino acid position were calculated as follows:

$$E=-\Sigma_{i=1}^{n}P_{i}\log_{b}p_{i}$$

where $P_{i}$ is the proportion of sequences that contain residue i at the codon in question, and b is typically base 2, Euler’s number e, or 10. Shannon Entropy-two analysis uses the natural logarithm log e.

### 2.5. Site-Specific Selection Analysis

PAL2NALv14 [24] was used to create a codon alignment based on protein multiple sequence alignment. The mixed-effects model of evolution (MEME) [25] implemented in the Datamonkey package (http://www.datamonkey.org/, accessed on 3 July 2022) was used to detect adaptive evolution. CD4 and CD8 T-cell epitope positions were retrieved from the Los Alamos National Laboratory website (http://hcv.lanl.gov/content/immuno/immuno-main.html, accessed on 3 July 2022) and the Immune Epitope Database and Analysis resource (http://www.iedb.org, accessed on 3 July 2022). We ensured that all HCV sequences were derived from DAA-naïve patients, excluding the reference sequences from DAA-treated patients described in the literature.

### 2.6. HCV Phylogenetic and Evolutionary Analysis

The Bayesian Markov chain Monte Carlo (MCMC) inference method, implemented in BEAST v1.10.4 (http://beast.community/tempest, accessed on 3 July 2022), was performed to estimate the ancestral relationships of the GT-3a, GT-3b, IDU, and BD subsets. The SRD06 nucleotide-partitioning model, an uncorrelated lognormal relaxed molecular clock, and a Bayesian Skyline coalescent model with 10 groups were employed. A lognormal prior distribution (mean, 0.001; standard deviation, 0.002) was used for the prior substitution rate to achieve MCMC convergence. To assess the sampling convergence of the MCMC procedures, the estimated effective sampling sizes (ESSs) were inspected (http://tree.bio.ed.ac.uk/software/tracer/, accessed on 3 July 2022). In this study, we considered sampling to be sufficient when all ESS values were ≥200. The trees from both chains were combined after removing the initial 10% burn-in. Maximum clade credibility trees were calculated and annotated using TreeAnnotator 1.10.4 (https://www.beast2.org/treeannotator/, accessed on 3 July 2022 and FigTree v1.4.1 (http://tree.bio.ed.ac.uk/software/figtree/, accessed on 3 July 2022).

### 2.7. Nucleotide Sequence Accession Numbers

The nucleotide sequences reported in this study were deposited in GenBank with the following accession numbers: OM896881-OM897000.

## 3. Results

### 3.1. Genotyping by Full-Length GT-3a and GT-3b Consensus Sequences

The predominant genotype/subtypes in the BD cohort were GT-1b (43.6%), followed by GT-6a (33.7%), GT-3a (9.5%), GT-3b (4.9%), GT-2a (7.3%), GT-1a (0.4%), GT-6e (0.4%), and GT-6k (0.2%) (Supplementary Table S2). Some of these data have been published previously [18]. By combining BD and IDU cohorts from previous studies [11,18], we found that subtypes GT-3a and GT-3b had gradually increased in recent years. In addition, there were significant differences in the distributions of the HCV genotype between the BDs and IDUs for GT-3b (Supplementary Table S2). The total numbers of HCV GT-3a and GT-3b samples from both cohorts were 75 and 76, respectively. Ultimately, 56 GT-3a and 64 GT-3b whole genome sequences were obtained by high-throughput sequencing (HTS); we did not generate any viral sequence data from 19 GT-3a and 12 GT-3b samples due to insufficient plasma volume.

A total of 54 GT-3a genomes had complete open-reading-frames, while the remaining 2 GT-3a sequences contained gaps. For the GT-3b sequences, 61 samples yielded full-length genomes, and the remaining 3 sequences had gaps (details shown in Supplementary Table S3 and Supplementary Figure S1). A total of 15 of the GT-3a sequences were derived from IDUs with a mean age of 35.15 ± 6.63 years, and 41 were from BDs with a mean age of 35.54 ± 6.57 years. For GT-3b, 43 sequences were from IDUs with a mean age of 34.09 ± 5.03 years, and 21 were from BDs with a mean age of 33.24 ± 7.08 years. Neither RDP4 nor GARD showed evidence of recombination for any of the GT-3a or GT-3b sequences. A maximum likelihood (ML) phylogenetic tree (Supplementary Figure S2) was estimated for all 120 sequences (GT-3a:56; GT-3b:64), including the incomplete genomes. The phylogenetic tree shows a topology similar to that estimated for the E1 and NS5B regions [11,18].

### 3.2. Comparative Analysis of HCV GT-3a and GT-3b Sequence Diversity

**Figure 1:**
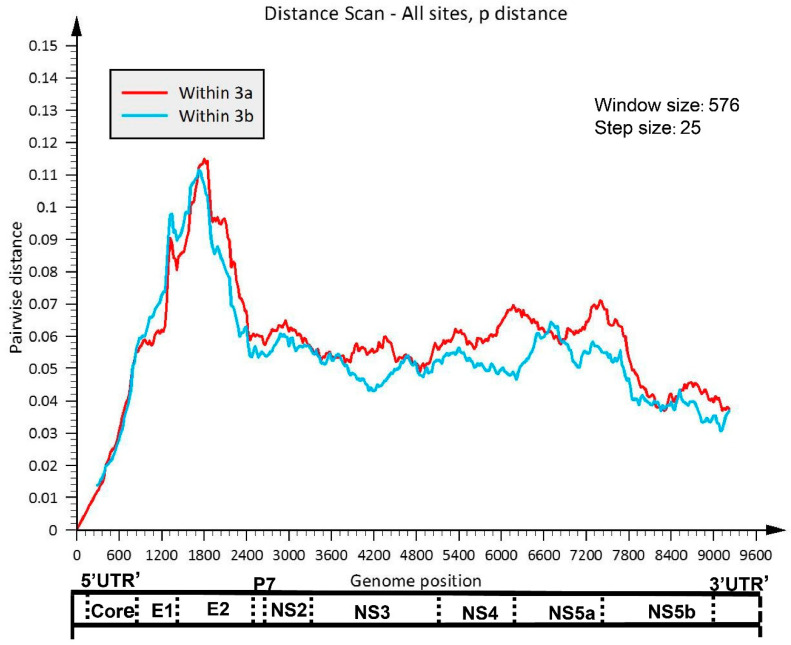
Full-genome sliding window plot for GT-3a and GT-3b nucleotide diversity (%). The GT-3a and GT-3b were plotted separately in color-coded solid lines.

**Figure 2:**
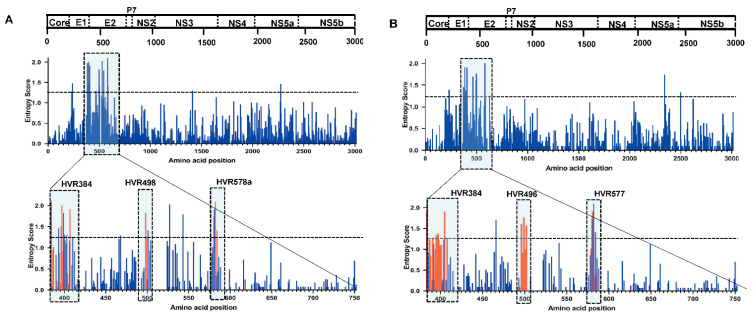
Sequence variability across the HCV subtype GT-3a and GT-3b genomes identifies three HVRs within E2. Each bar represents variability at a single amino acid site. The genomic region of each encoded protein is indicated at the top of the figure. Positively selected sites are highlighted in red bars. The entropy score at each amino acid site in E2 for HCV GT-3a (**A**) and GT-3b (**B**) is shown.

SSE p-distance analysis illustrated that the pairwise distance along the genome of the GT-3a sequences did not completely align with GT-3b (Figure 1). For GT-3b, there was a slightly higher diversity in the coding regions for E1 and the N-terminal portion of E2; elsewhere, diversity was marginally lower in the coding regions for the non-structural proteins when compared with GT-3a. Shannon Entropy–two analysis evaluated the sequence diversity of the full-length sequences from GT-3a and GT-3b subsets. The results showed that the regions of highest variability (defined here as entropy scores of >1.25) were predominantly located in the coding regions for the envelope proteins, particularly E2 (Figure 2, upper panels). Further analysis of the E2 region revealed three distinct regions of genomic variability, including hyper-variable region 1 (HVR1; named HVR384 in Figure 2, lower panels), which is known to be present in all HCV genotypes except GT-3 [26]. For GT-3a, according to the first location of amino acids for which the entropy scores were >1.25, the second HVR was named HVR498; it represents amino acids 498 to 504, and is seven residues in length. The third region in E2 with variability was named HVR578a, which spans amino acids 578a to 579 and is 6 residues long (Figure 2A). An insertion of 5 amino acids is found in HVR578a and lies at a putative N-linked glycosylation site (N-X-S, where X represents any amino acid except proline, N represents asparagine, and S represents serine) (Supplementary Figure S3A). For GT-3b, the second and third HVR regions displayed slight differences in both amino acid location and length of the HVR region (for the third HVR region). These were HVR496 (spans amino acids 496–502 and is 7 amino acids long) and HVR577 (spans amino acids 577–579 and is 8 amino acids long; Figure 2B). Similar to the equivalent region in GT-3a, HVR577 in the GT-3b E2 region contains a 6 amino acid insertion; this segment also encodes a putative N-linked glycosylation site N-X-T (T represents threonine; Supplementary Figure S3B). In theory, regions of high variability may arise because selective forces induce variation. Evolutionary analysis of E2 by MEME revealed that almost 50% of the positively selected sites were located almost exclusively within the 3 HVR regions in the GT-3a and GT-3b sequences (Figure 2, lower panels).

### 3.3. The Origin and Epidemiology of GT-3 Worldwide

**Figure 3:**
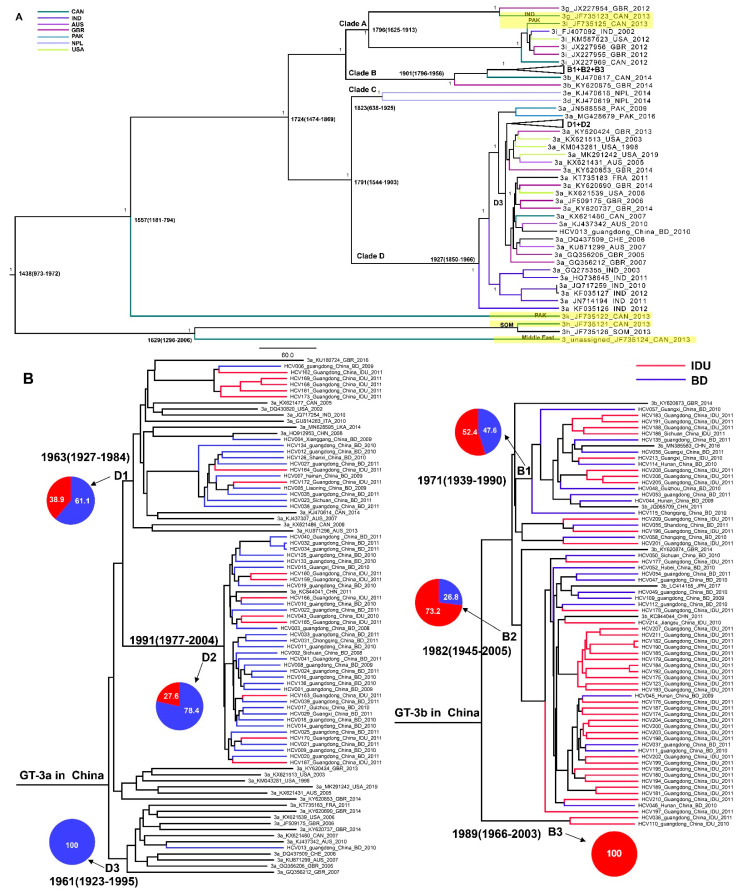
Bayesian phylogenetic tree based on full-length sequences of HCV GT-3 isolates. The posterior value and tMRCA are shown for the main clusters. References are named with the following format: Subtype_GenBank accession number_collection country_collection date. (**A**) Bayesian phylogenetic tree with collapse of GT-3a and GT-3b in China. The yellow highlights showed the origin of reference sequences. (**B**) The clade of GT-3a and GT-3b in China and the pie chart which shows the proportions of IDUs/BDs per each group.

The maximum clade credibility (MCC) phylogeny of HCV GT-3 was obtained from - Bayesian molecular clock analysis of the 59 reference sequences and 120 sequences in this study (Figure 3). The evolution rate of HCV GT-3 was 1.280E-3 (6.139E-4, 2.026-3) substitutions/site/year (s/s/y) with a most recent common ancestor (tMRCA) dated from 1438 (95% HPD: 973, 1972) based on all GT-3 lineages. Two GT-3h and the unassigned variant JF735124 sequences yielded the oldest lineage for GT-3, with a common node at 1629 (95% HPD: 1296, 2006); these variants originated in the Middle East and Africa [27]. Other reference subtypes combined with the sequences in this study formed four clades named A, B, C, and D, which separated from year 1724 (95% HPD: 1474, 1869). The GT-3g and GT-3i reference sequences grouped in Clade A, and all GT-3b isolates (references and sequences in this study) formed Clade B. Clade C only contained two isolates (GT-3d and GT-3e) that were both from Nepal. Clade D contained only GT-3a sequences. Geographically, all GT-3a sequences had their origins in India, which were the root of GT-3a in Clade D, transmitted from an ancestor of the India subcontinent to South Asia, Europe, North America, and then to East Asia (Figure 3A).

### 3.4. The Origins of GT-3a and GT-3b and the Transmission Relationship between IDUs and BDs in China

The evolution rate of HCV GT-3a was 1.391E-3 (1.075E-3, 1.720E-3) s/s/y, which was marginally higher than HCV GT-3b in China (1.343E-3 [9.908E-4, 1.706E-3]) s/s/y. The GT-3a sequences in the MCC tree (shown in Figure 3B) formed three groups (D1, D2, and D3), indicating that there are three transmission routes across China, especially in Guangdong Province in both IDU and BD populations. Only one sequence (HCV013) in D3 may be a chance spread event from North America and Europe. The majority (67.3%) of our GT-3a sequences originated from 1991 (95% HPD: 1977, 2004), forming a Chinese-specific cluster named Group D2. Our study’s remaining sequences (D1) originated from the year 1963 (95% HPD: 1927, 1984) and therefore were earlier than D2. We speculate that subclade D3 was globally distributed while D2 sequences were perhaps only endemic in China. Furthermore, D1 sequences contained a higher proportion of IDUs than D2, suggesting that GT-3a in D1 was transmitted from IDUs to BDs. The GT-3b sequences in our study formed three groups named B1, B2, and B3 (shown in Figure 3B). It seems that the GT-3b sequences originated from the IDU population as two IDU sequences (HCV036 and HCV110) were placed at the root of GT-3b in Chinese sequences. Thereafter, GT-3b, through the mutual transmission between BDs and IDUs, could ultimately have formed as an independent endemic in IDUs according to the topology of B2. B2 and B1 have the common ancestor that separated from B3.. The isolates in B1 were mutual transmissions between IDUs and BDs.

### 3.5. The Effective Number of HCV Infections in China

**Figure 4:**
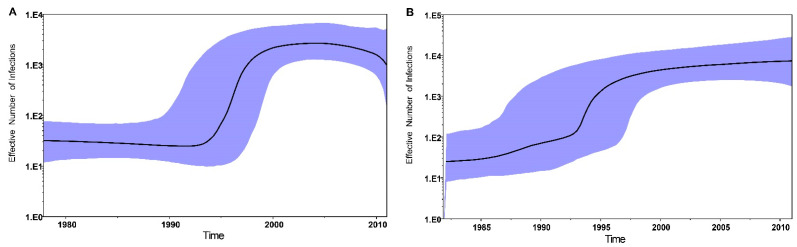
Bayesian skyline plot showing the predicted epidemic history of HCV GT-3a (**A**) and GT-3b (**B**) in China. The solid black line represents the estimated effective number of infections over time. The blue area indicates the 95% highest posterior density confidence intervals for this estimate.

The reconstructions of the epidemics for GT-3a and GT-3b in China are shown on the Bayesian skyline plots in Figure 4. The plots represent the effective numbers of HCV infections in China over time, back to the estimated tMRCA of GT-3a and GT-3b. The effective number of GT-3a infections in China experienced exponential growth from 1993 to 2005; nevertheless, after 2005, the effective number of GT-3a infections gradually decreased over time. For GT-3b in China, the effective numbers gradually increased over time except for a rapid, short-term increase from 1993 to 1996.

### 3.6. The Positive Selection Sites in HCV GT-3a and GT-3b Sequences in China

**Figure 5:**
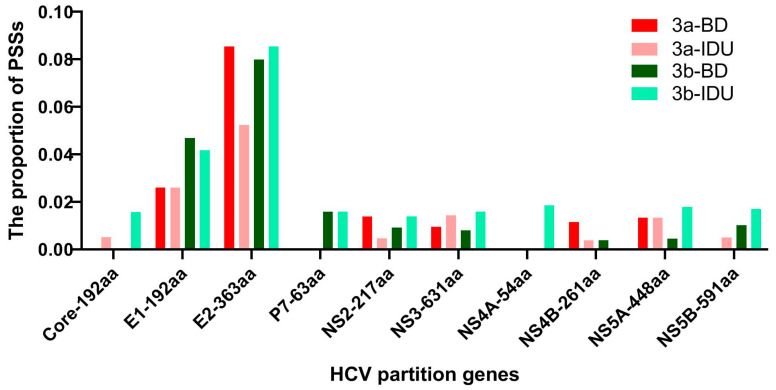
The distribution of PSSs in the polyprotein of the H77 reference sequence for HCV GT-3a-BDs, GT-3a-IDUs, GT-3b-BDs, and GT-3b-IDUs. PSS indicates a positively selected site.

**Figure 6:**
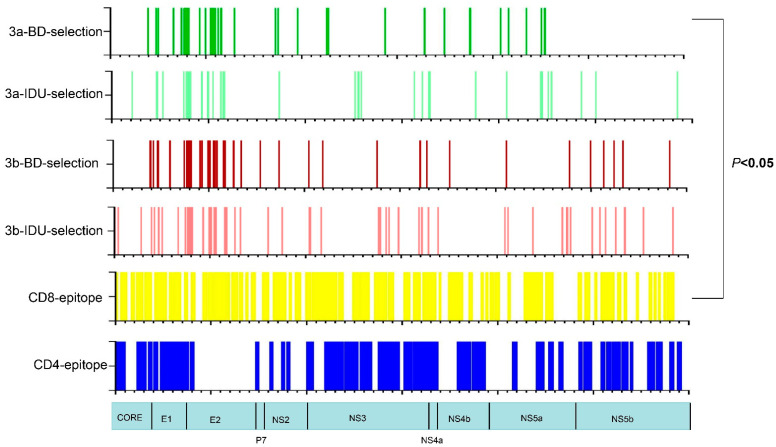
Map of the HCV GT-3a-BDs (Dark green), GT-3a-IDUs (Light green), GT-3b-BDs (Dark red), and GT-3b-IDUs (light red), indicating the locations of PSSs, CD8 T cell epitopes (yellow), and CD4 T cell epitopes (blue).

**Figure 7:**
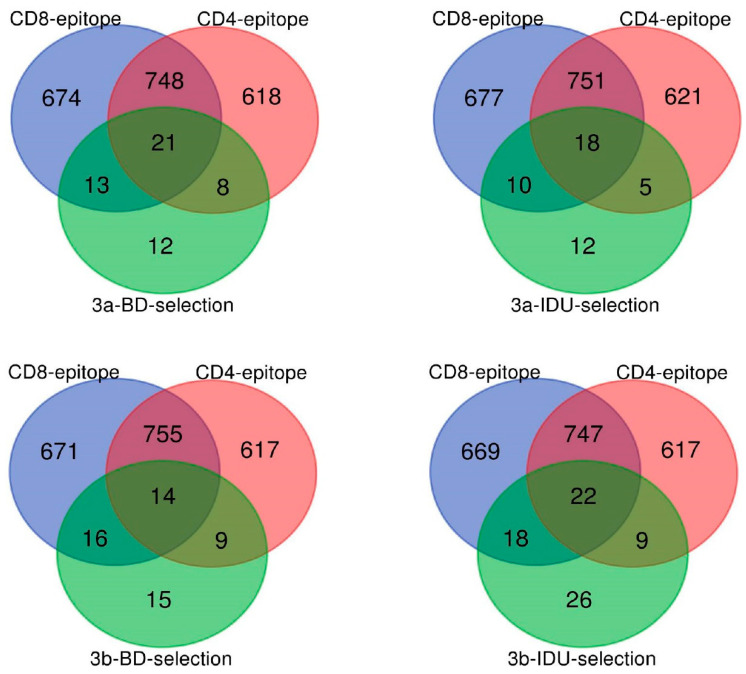
Venn diagrams for the overlap of the CD8/4 epitopes with the selection in HCV GT-3a-BDs, GT-3a-IDUs, GT-3b-BDs4, and GT-3b-IDUs.

Positive selection was found at 54 amino acid sites for GT-3a-BDs, 45 amino acid sites for GT-3a-IDUs, 55 amino acid sites for GT-3b-BDs, and 75 amino acid sites for GT-3b-IDUs. The protein coding regions with the highest proportions of positively selected sites (PSSs) were E1 and E2 in these four populations (Figure 5), which accounted for 66.7% (36/54) among all regions in GT-3a-BDs, 69.1% (38/55) in GT-3b-BDs, 53.0% (24/45) in GT-3a-IDUs, and 52.0% (39/75) in GT-3b-IDUs. Twenty-one PPSs were found to be positively selected in both GT-3a-BDs and GT-3b-BDs, which are located in E1 (one site), E2 (thirteen sites), NS3 (two sites), NS4B (one site), and NS5A (four sites). Twenty-seven PPSs were found in both GT-3a-IDUs and GT-3b-IDUs, which are located in E1 (five sites), E2 (twenty-one sites), and NS3 (one site). A map of the different populations representing the different layers of data analyzed (PSSs, CD8 and CD4 T cell epitopes) is shown in Figure 6. A Venn diagram was performed for the overlap of the CD8/4 epitopes with selection in the four populations (Figure 7). An association between PSS and the presence of CD8 epitopes was found (χ^2^ = 9.675, p < 0.05), i.e., CD8 T cell epitopes tended to be under negative selection in a GT-3a-BDs population. There were no associations between PSS, CD8 T cell epitopes, and CD4 T cell epitopes in GT-3a-IDUs, GT-3b-BDs, and GT-3b-IDUs. Four amino acid sites within the CD8 T cell epitopes were positively selected in four populations. Among these, site 372 is located in the E1 region, which is targeted by cytotoxic T lymphocyte (CTL)-restricted HLA type A*02:01. Amino acids 398, 399, and 401 are located in the E2 region, which is also targeted by HLA type A*02:01.

## 4. Discussion

In this study, we employed HTS to successfully obtain 120 HCV GT-3a and GT-3b genomes for estimating the origin and divergence of HCV GT-3 worldwide and analyzing the relationship between HCV transmission route and selection pressure between IDUs and BDs in China. This is the first time that a substantial number of HCV GT-3b full-length genomes have been obtained, since only 14 full-length HCV GT-3b genomes are presently archived in public databases.

HCV is a highly diverse pathogen at the nucleotide sequence level; the extent of this diversity varies across the genome and is dependent on the structure and function of its encoded proteins [28]. Genome-wide diversity patterns were similar in the GT-3a and GT-3b subtypes, although we did note slightly higher diversity in the coding regions for E1 and the N-terminal portion of E2 in GT-3b. Elsewhere, diversity was marginally lower in the coding regions for the non-structural proteins for GT-3b compared with GT-3a. In agreement with a previous study [29], the highest diversity regions of GT-3a and GT-3b were distributed in E1 and E2, followed by NS2, NS4A/B, and NS5A. By contrast, low diversity values were obtained in core and NS5B. E1 and E2 encode the envelope glycoproteins, which are targeted by the neutralizing antibody response of the host [30]. Owing to the large plasticity in the highly variable regions in these two proteins [31], the virus is capable of escaping from neutralizing antibodies upon transmission to a new host. In this study, three HVRs were found in the E2 region in both subtypes, which is consistent with a previous GT-3a study [26]. The boundaries of HVR2 and HVR3 were slightly different from the previous GT-3a study; the same situation was also found in GT-3b. Almost 50% of positive selection sites were concentrated within the three HVR regions in the GT-3a and GT-3b, supporting the theory that a highly diverse protein should coincide with a large number of positive selected sites [29]. Low diversity positions (shown in Figure 2) in HVRs play an important role in maintaining the structures of HVR domains [32]. In terms of function, HVR1 contributes to virus escape by acting as a decoy antigen that diverts the host immune response away from more conserved neutralizing epitopes [33]. It can also modulate the neutralization potential of monoclonal antibodies. Thus, HVR1 can obstruct the viral CD81 binding site, conceal the neutralization epitopes of E2 protein, and reduce the neutralization activity of E2 antibody [34,35]. HVR2 can influence antibody recognition of the E2 glycoprotein and may contribute to immune evasion [34]. No information is available on the impact of HVR3 on antigenicity, which should be the focus of further studies. However, there is a putative N-linked glycosylation site within HVR3 both in GT-3a and GT-3b that has been shown to be involved in correct folding and the formation of E1/E2 complexes [36]. Subtype GT-1a has a glycosylation site in close proximity to HVR578a/577, suggesting a critical role for glycosylation in this region.

From our analysis, the tMRCA of GT-3 may date back to 581 years ago (tMRCA:1438), which is older than a previous estimate of 457.8 years (95% credible region, 350.6 to 587.5) ago [27]. This discrepancy may be attributed to the larger number of full-length sequences included in our study. However, accurately timing the tMRCA for HCV genotypes is liable to be subject to considerable inaccuracy since viral sequences have been determined from samples collected in the recent past. In addition, we found that the origins of GT-3 may trace back to Africa, which is generally coincident with the results from Chunhua Li et al. [27], as GT-3h and the unassigned variant represent the oldest GT-3 isolates from individuals in Africa and the Middle East. These authors speculated that the ancestral GT-3 strains had spread to South Asia from Africa, which was supported by the ML tree’s topology in our study. GT-3k reference isolates from South Asia were the descendants of GT-3h and unassigned variant JF735124 sequences, while it appears that GT-3k is the ancestor of other GT-3 subtypes. The presence of a high prevalence of HCV GT-3 with the coexistence of different subtypes of GT-3 in India suggests that India is a probable site for the emergence of HCV genotype 3 [37], discussed by Zehender et al. [38], which supported our above results. Our data suggest that HCV GT-3a was transmitted from the Indian subcontinent to Europe and then to Asia around 1927 (1850–1966). However, this dating should be interpreted with caution given the limited number of GT-3a sequences included in our study. Nonetheless, this time frame corresponds to World War I and - British colonial period in India. The origin of GT-3a has also been examined in previous studies for different geographic regions with full-length genomes [39,40]. In North America and Australia, subtype GT-3a originated at around 1950, while in India, GT-3a sequences appeared and later dispersed to the United Kingdom around the mid-1940s. A study using 42 sequences of 8 distinct GT-3 subtypes estimated the tMRCA to be around 1934 (95% HPD, 1915–1949) for GT-3a [27]. Interestingly, in these previous studies, the first sequences to branch out from the root were from India, which is consistent with our study. GT-3b sequences coalesced in 1901(1796–1956) (95% HPD: 1796, 1956). There is no putative origin for GT-3b since so few full-length reference sequences are available.

The evolution rate of HCV GT-3a (1.391E-3 s/s/y) was slightly higher than that of GT-3b (1.343E-3 s/s/y) in China, and similar to the evolution rates of GT-3a from a global data set (1.65E-3 s/s/y) [41].Concomitantly, the dated origin of GT-3a (1962) was slightly earlier than that for GT-3b (1970) in China, and also earlier than 1987, the year GT-3b emerged in China by partial gene analysis in a previous study [12]. A study from Pakistan depicted the dispersal of GT-3b from Pakistan to China in the early 19th century [42]. We speculate that the different regions of HCV used to perform BEAST analysis and the geographic locations contributed to this discrepancy. In this study, we utilized whole genome sequences of HCV to perform a Bayesian analysis, which was considered more precise and reliable than analyses performed with partial regions. HCV GT-3b may trace back to Thailand according to coalescent analysis by partial gene analysis (NS5B and E1) [12]. However, so few GT-3b whole genomes are archived in the database, we cannot speculate on the origin of this subtype worldwide.

Our study suggests three transmission routes across China related to GT-3a, both in IDUs and BDs. One of the major transmission routes of GT-3a was restricted to China and formed a Chinese-specific cluster both in BDs and IDUs, which indicates that GT-3a could have been transmitted by IDUs and then become endemic in the general population. For GT-3b, our study reveals that this subtype might have originated from IDUs in China. Thereafter, the mutual transmission between BDs and IDUs ultimately formed an independent endemic spread in IDUs. Previous studies have also shown that GT-3 in IDUs spreads to the general population by sharing infected equipment and through high-risk sexual behaviors [43,44]. The Bayesian skyline plots for GT-3a and GT-3b reveal an exponential growth of infections in China between 1993 and 2005. Soon after governmental reform and the open-door policies of the 1980s, the use of injectable drugs became popular in China and was accompanied by heroin abuse [45]. A study showed that the number of registered drug users in China increased from 70,000 in 1990 to 1.16 million by the end of 2005, and intravenous injection accounted for 50–70% of this figure [46]. These data are consistent with the projections of trends in expansion in GT-3a and GT-3b infections. Moreover, the BSP curve showed that the effective numbers of GT-3a infections have gradually decreased after 2005, which corresponds to the findings in this study that the transmission of GT-3a has shifted from IDUs to the general population. Conversely, the effective number of GT-3b infections has gradually increased over time, which may result from GT-3b continuing to circulate in IDUs.

In total, 1.49–2.49% of full-genome codon positions were positively selected, which is higher than in a previous study (0.53%) [29]. The likely reason for the varied proportion of PPSs was the analysis method used, utilizing the two-rate fixed effects likelihood (FEL) and single-likelihood ancestor counting (SLAC). In our study, we used the MEME method, which has a superior performance to that of FEL and SLAC under a broad range of scenarios. The proportion of PPSs in GT-3a and GT-3b was less than GT-6a in our previous study [47], probably explained by the phenomenon that HCV GT-6a was more prevalent than either of the GT-3 subtypes in Guangdong Province. The number of PPSs in GT-3b was higher than in GT-3a, suggesting that GT-3b has a stronger potential ability to escape host immune pressure. In recent years, injectable drugs have become the predominant transmission route of HCV infection in China. Therefore, preventive education about HCV transmission and periodic testing should be considered. In addition, the majority of PPSs were located in the E1 and E2 regions both in BDs and IDUs, which is concordant with their functional roles in viral escape from immunological responses [48]. However, the proportion of PPSs in E1 and E2 in IDUs (99.3%, 26/27) was higher than in BDs (66.7%, 14/21), i.e., different transmission routes impact the distribution of PPSs among HCV genomes. The higher proportion of PPSs in E1 and E2 in IDUs indicated that IDUs were more able to escape the host immunity as neutralizing antibodies target E1 and E2. The fact that CD8 T cell epitopes tend to be under negative selection in a GT-3a-BDs population showed that other populations are prone to HCV infection. Four amino acid sites within CD8 T cell epitopes were positively selected in four populations. They were all located in epitopes targeted by HLA type A*02:01, accounting for 12.8% of the population in Guangdong Province (http://www.allelefrequencies.net/ accessed on 3 July 2022).

## 5. Conclusions

In conclusion, we have analyzed the degree of genetic variability and the origin of HCV GT-3 worldwide using GT-3a and GT-3b whole genome sequences from IDUs and BDs in China and reference sequences from various countries. Three HVRs were found in both GT-3a and GT-3b subsets for the E2 glycoprotein genes. HCV GT-3a was transmitted from the Indian subcontinent to South Asia, Europe, and North America and then became endemic in China. In addition, in China, GT-3a may have been transmitted by IDUs and become endemic in the general population, while GT-3b may have originated from IDUs and then underwent mutual transmission between BDs and IDUs and ultimately formed an independent endemic spread in IDUs. The GT-3a and GT-3b sequences from BDs and IDUs exhibit different selective pressures: The proportion of PPSs in the E1 and E2 coding regions in IDUs was higher than in BDs, whereas the number of PPSs was higher in GT-3b and IDUs. These results potentially provide information about the interactions between transmission route and host immune pressure and can help us formulate an HCV GT-3a and GT-3b prevention strategy in China.

## Data Availability

Data are contained within the article or Supplementary Materials. Further inquiries can be directed to the corresponding author/s.

## Supplementary Materials

The following supporting information can be downloaded at: https://www.mdpi.com/article/10.3390/v14071514/s1, Figure S1: The average read depth (black dot), minimum depth (lower limit) and maximum depth (upper limit) for each sample in this study. Figure S2: Phylogenetic tree of all HCV reference sequences and GT-3a and GT-3b sequences in this study. The HCV genotypes (gt1–gt8) are labeled on the branches. Sequences labeled with red and green solid circles represent GT-3a (n = 56) and GT-3b (n = 64) sequences, respectively, from this study. Bootstrap support values are only shown for the major clades. References are named with the following format: subtype_accession number. Figure S3: The amino acid sequence of HVR578a within GT-3a and HVR577 within GT-3b. The 5-amino-acid insertion for GT-3a and 6-amino-acid insertion for GT-3b are shown with a bar. The E2 of H77 1a sequence is shown for comparison. Table S1: Details of HCV GT-3 reference sequences (n = 59) used for analysis. Table S2: The HCV genotype distribution between BDs and IDUs; Table S3: Consensus calling results of GT-3a and GT-3b samples.
