# Supplementary material for: The Transmission Route and Selection Pressure in HCV Subtype 3a and 3b Chinese Infections: Evolutionary Kinetics and Selective Force Analysis
Source: Viruses. 2022 Jul 11;14(7):1514. doi: 10.3390/v14071514 (PMC9324606; doi:10.3390/v14071514)
Supplement: Supplementary file 1 [file viruses-14-01514-s001.zip › viruses-1726694 (Table S1-S3, Figure S1 and S3).pdf]

Supplementary Materials

Table S1. Details of HCV GT-3 reference sequences (n=59) used for analysis.

Subtype	Accession Number	Collection_Country	Collection_date	Source of sample from non-collection_country
3	JF735124	CAN	2013	Middle-east
3a	DQ430820	USA	2002	
3a	DQ437509	CHE	2006	
3a	GQ275355	IND	2003	
3a	GQ356206	GBR	2005	
3a	GQ356212	GBR	2007	
3a	JN714194	IND	2011	
3a	GU814263	ITA	2010	
3a	HQ738645	IND	2011	
3a	HQ912953	CHN	2008	
3a	JF509175	GBR	2006	
3a	JN588558	PAK	2009	
3a	JQ717254	IND	2010	
3a	JQ717259	IND	2010	
3a	KC844041	CHN	2011	
3a	KF035126	IND	2012	
3a	KF035127	IND	2012	
3a	KJ437307	AUS	2007	
3a	KJ437342	AUS	2010	
3a	KJ470614	CAN	2014	
3a	KM043281	USA	1998	
3a	KT735183	FRA	2011	
3a	KU180724	GBR	2016	
3a	KU871296	AUS	2013	
3a	KU871299	AUS	2007	
3a	KX621431	AUS	2005	
3a	KX621477	CAN	2005	
3a	KX621480	CAN	2007	
3a	KX621486	CAN	2009	
3a	KX621513	USA	2003	
3a	KX621539	USA	2006	
3a	MG428679	PAK	2016	
3a	MK291242	USA	2019	
3a	KY620424	GBR	2013	
3a	KY620690	GBR	2014	
3a	KY620737	GBR	2014	
3a	KY620853	GBR	2014	
3a	KY620873	GBR	2014	
3b	MN385583	CHN	2016	
3b	MN628595	LKA	2014	
3b	JQ065709	CHN	2011	
3b	KC844044	CHN	2011	
3b	KJ470617	CAN	2014	
3b	LC414155	JPN	2017	Individual from China

3b	KY620874	GBR	2014	
3b	KY620875	GBR	2014	
3d	KJ470619	NPL	2014	
3e	KJ470618	NPL	2014	
3g	JF735123	CAN	2013	Individual from India
3g	JX227954	GBR	2012	
3h	JF735121	CAN	2013	Individual from SOM
3h	JF735126	SOM	2013	
3i	FJ407092	IND	2013	
3i	JF735125	CAN	2013	Individual from PAK
3i	JX227955	GBR	2012	
3i	JX227956	GBR	2012	
3i	11JX227969	CAN	2012	
3i	KM587623	USA	2012	
3k	JF735122	CAN	2013	Individual from PAK

CHN: China; CAN: Canada; AUS: Australia; USA: United States; CHE: Switzerland; IDN: Indonesia; GBR: United Kingdom; ITA: Italy; PAK: Pakistan; FRA: France; JPN: Japan; NPL: Nepal; LKA: Sri Lanka;

Table S2. The HCV genotype distribution between BDs and IDUs.

HCV genotypes	BDs n (%)	IDUs* n (%)	χ^2	P
HCV 1a	2(0.40)	7(2.85)	8.121	<0.05
HCV 1b	215(43.61)	14(5.37)	1.3103E2	<0.001
HCV 2a	36(7.30)	0(0.00)	18.88	<0.001#
HCV 3a	47(9. 53)	28(11.38)	0.615	0.433
HCV 3b	24(4.87)	52(21.14)	40.29	<0.001
HCV 6a	166(33.67)	142(57.72)	39.06	<0.001
HCV 6e	2(0.41)	2(0.81)	-	-
HCV 6k	1(0.20)	1（0.41））	-	-
Total	493(100)	246(100)	-	-

*: These data was from previously study. #: Fisher exact test.

Table S3. Consensus calling results of GT-3a and GT-3b samples.

Samples name	Average depth	Min_depth	Max_depth	Coverage(%)	Phred qulity score	Method	Subtype	Length of sequences >9000pb	Gap position	Gap Long
HCV01	423	5	758	100	35	reference	3a	YES		
HCV02	9541	5	20377	100	35	reference	3a	YES		
HCV03	359	5	704	100	35	reference	3a	YES		
HCV04	440	5	1021	100	35	reference	3a	YES		
HCV05	95	5	254	100	35	De novo assembly	3a	YES		
HCV06	3075	5	6817	100	35	reference	3a	YES		
HCV07	5980	5	13604	100	35	reference	3a	YES		
HCV08	3285	5	6523	100	35	reference	3a	YES		
HCV09	1946	5	3760	100	35	reference	3a	YES		

HCV10	1147	5	2147	100	35	reference	3a	YES
HCV11	4698	5	9562	100	35	reference	3a	YES
HCV12	3152	5	6739	100	35	reference	3a	YES
HCV13	48	5	127	99	35	reference	3a	YES
HCV14	5037	5	9944	100	36	reference	3a	YES
HCV15	2352	5	4990	100	34	reference	3a	YES
HCV16	3707	8	6951	100	35	reference	3a	YES
HCV17	1449	5	2439	100	35	reference	3a	YES
HCV18	893	5	1627	100	35	reference	3a	YES
HCV019	275	5	6329	100	34	reference	3a	YES
HCV020	1581	10	3219	100	34	reference	3a	YES
HCV021	3476	5	6388	100	35	reference	3a	YES
HCV022	279	5	13312	100	36	reference	3a	YES
HCV023	1059	5	2134	100	35	reference	3a	YES
HCV024	504	5	1126	100	35	reference	3a	YES
HCV025	13343	1448	58262	100	35	reference	3a	YES
HCV027	1229	5	3488	100	34	reference	3a	YES
HCV029	725	5	1558	100	35	reference	3a	YES
HCV031	1166	5	22334	100	35	reference	3a	YES
HCV032	1764	5	3995	100	35	reference	3a	YES
HCV033	4202	9	7797	100	35	reference	3a	YES
HCV034	2568	5	5430	100	35	reference	3a	YES
HCV035	810	5	2176	100	35	reference	3a	YES
HCV038	40	5	91	100	35	reference	3a	YES
HCV039	2401	5	4614	100	35	reference	3a	YES
HCV040	6826	5	13843	100	35	reference	3a	YES
HCV041	15680	5	27289	100	35	reference	3a	YES
HCV043	1412	29	3031	100	35	reference	3a	YES
HCV125	15270	5	34270	100	35	De novo assembly	3a	YES
HCV126	16681	5	37156	100	37	De novo assembly	3a	YES

HCV133	3729	161	9674	100	37	De novo assembly	3a	YES		
HCV134	28195	5	71730	100	35	De novo assembly	3a	YES		
HCV136	84608	5	196967	100	35	De novo assembly	3a	YES		
HCV159	55959	5	175613	100	35	De novo assembly	3a	YES		
HCV160	31304	5	106504	100	35	De novo assembly	3a	YES		
HCV161	2631	5	18766	100	35	reference	3a	YES		
HCV162	42899	5	369286	98	36	reference	3a	NO	Core/E2/NS2/NS3/NS5B	660
HCV163	60535	5	219405	100	35	reference	3a	YES		
HCV164	17310	5	88237	94	35	reference	3a	NO	Core//E1/E2/NS2/NS3/NS4B/NS5B	832
HCV165	95484	5	292283	100	35	reference	3a	YES		
HCV166	78257	5	267746	100	35	reference	3a	YES		
HCV167	245604	5	831272	100	35	reference	3a	YES		
HCV168	292549	5	1000114	100	35	reference	3a	YES		
HCV169	44898	5	198739	100	37	reference	3a	YES		
HCV170	97685	5	333862	100	39	reference	3a	YES		
HCV172	154188	5	1170162	100	35	reference	3a	YES		
HCV173	22934	5	104079	100	36	reference	3a	YES		
HCV036	396	5	737	100	35	De novo assembly	3b	YES		
HCV037	7152	5	14310	100	35	De novo assembly	3b	YES		
HCV044	3259	5	7330	100	35	De novo assembly	3b	YES		
HCV045	20	5	71	99	35	De novo assembly	3b	YES		
HCV046	4194	5	8425	100	35	De novo assembly	3b	YES		
HCV047	1105	5	1934	100	35	De novo assembly	3b	YES		
HCV048	1562	5	3167	100	35	De novo assembly	3b	YES		
HCV049	2827	5	5739	100	35	De novo assembly	3b	YES		
HCV050	3486	5	7080	100	35	De novo assembly	3b	YES		

HCV052	2423	5	5676	100	36	De novo assembly	3b	YES		
HCV053	7294	5	14070	100	35	De novo assembly	3b	YES		
HCV054	7553	5	15006	100	35	De novo assembly	3b	YES		
HCV055	5602	5	11498	100	35	De novo assembly	3b	YES		
HCV056	1160	5	2312	100	35	De novo assembly	3b	YES		
HCV057	7	5	33	99	35	De novo assembly	3b	NO	Core/E1/E2/P7/NS2/NS3/NS5B	2248
HCV058	2027	13	3756	100	35	De novo assembly	3b	YES		
HCV109	61	5	448	100	37	De novo assembly	3b	YES		
HCV110	6816	5	83256	100	37	De novo assembly	3b	YES		
HCV111	1968	5	24349	100	37	De novo assembly	3b	YES		
HCV112	999	78	13315	100	37	De novo assembly	3b	YES		
HCV114	1510	5	13312	100	37	De novo assembly	3b	YES		
HCV115	6	5	43	96	27	De novo assembly	3b	NO	Core/E1/E2/P7/NS2/NS3/NS4A/NS5B	4823
HCV123	101	5	833	99	37	De novo assembly	3b	YES		
HCV135	13343	1448	58262	100	35.00	De novo assembly	3b	YES		
HCV174	28255	5	140184	100	35	De novo assembly	3b	YES		
HCV175	10551	5	104043	100	36	De novo assembly	3b	YES		
HCV176	15792	5	101949	100	36	De novo assembly	3b	YES		
HCV177	21444	5	178650	98	35	De novo assembly	3b	NO	Core/E1/NS3/NS5B	959
HCV178	85535	5	466150	100	35	De novo assembly	3b	YES		
HCV179	11994	5	110866	100	35	De novo assembly	3b	YES		
HCV180	17817	5	82176	100	35	De novo assembly	3b	YES		
HCV181	26174	6	126336	100	35	De novo assembly	3b	YES		
HCV182	12945	5	83096	100	35	De novo assembly	3b	YES		
HCV183	23543	5	113049	100	35	De novo assembly	3b	YES		
HCV184	6509	5	56739	100	35	De novo assembly	3b	YES		

HCV185	7751	5	81270	100	35	De novo assembly	3b	YES
HCV186	14717	5	119946	100	35	De novo assembly	3b	YES
HCV187	11102	5	67342	100	36	De novo assembly	3b	YES
HCV188	23940	583	137921	100	35	De novo assembly	3b	YES
HCV189	34715	5	115818	100	35	De novo assembly	3b	YES
HCV190	2477	5	13633	100	35	De novo assembly	3b	YES
HCV191	33525	2704	154329	100	35	De novo assembly	3b	YES
HCV192	11818	5	104341	100	35	De novo assembly	3b	YES
HCV193	38675	5	252317	100	35	De novo assembly	3b	YES
HCV194	45252	5	274043	100	35	De novo assembly	3b	YES
HCV195	44984	5	237921	100	35	De novo assembly	3b	YES
HCV196	12532	5	71587	100	35	De novo assembly	3b	YES
HCV197	17716	5	85822	100	35	De novo assembly	3b	YES
HCV198	41161	5	177560	100	35	De novo assembly	3b	YES
HCV199	21396	5	84005	100	35	De novo assembly	3b	YES
HCV200	7760	5	78414	98	35	De novo assembly	3b	YES
HCV201	30831	5	174223	100	35	De novo assembly	3b	YES
HCV202	29642	5	249234	100	35	De novo assembly	3b	YES
HCV203	6562	5	64085	100	35	De novo assembly	3b	YES
HCV204	33747	5	145236	100	35	De novo assembly	3b	YES
HCV205	35945	5	242566	100	35	De novo assembly	3b	YES
HCV206	77333	5	474392	100	35	De novo assembly	3b	YES
HCV207	26904	5	120810	99	35	De novo assembly	3b	YES
HCV208	25695	3126	128129	100	35	De novo assembly	3b	YES
HCV209	18044	683	125326	100	35	De novo assembly	3b	YES
HCV210	51681	5	455103	100	35	De novo assembly	3b	YES

HCV211	21280	5	194954	100	35	De novo assembly	3b	YES
HCV213	2725	344	15342	100	35	De novo assembly	3b	YES
HCV214	14273	2193	78561	100	35	De novo assembly	3b	YES

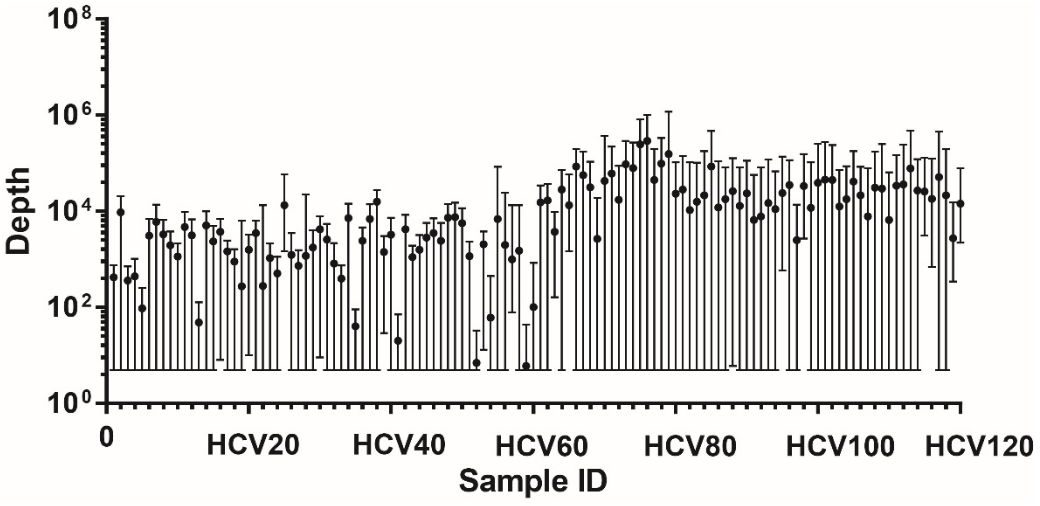

Figure S1. The average read depth (black dot), minimum depth (lower limit) and maximum depth (upper limit) for each sample in this study.

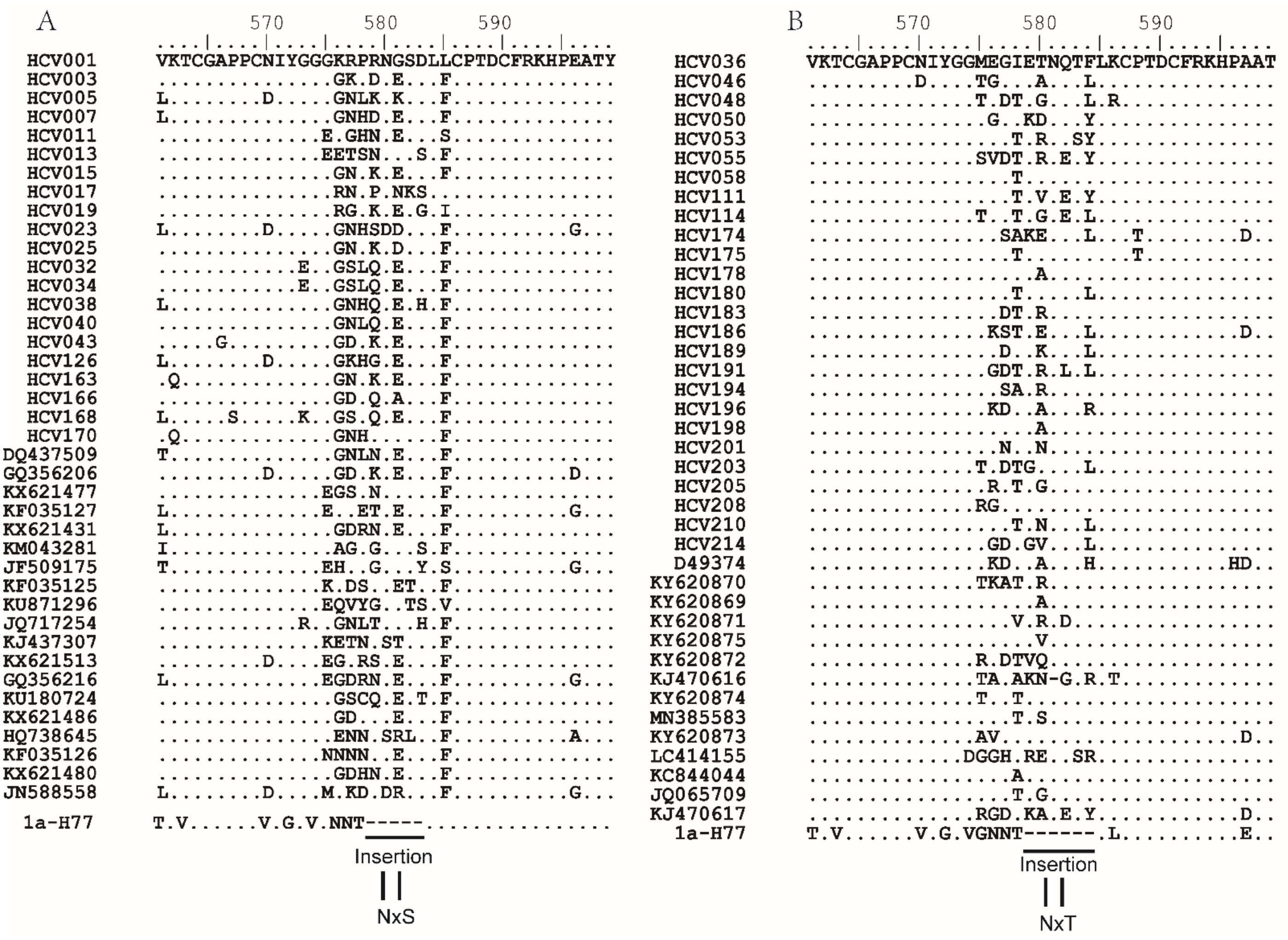

Figure S3. The amino acid sequence of HVR578a within GT-3a and HVR577 within GT-3b. The 5-amino-acid insertion for GT-3a and 6-amino-acid insertion for GT-3b are shown with a bar. The E2 of H77 1a sequence is shown for comparison.
