# Supplementary figures and images for: The Transmission Route and Selection Pressure in HCV Subtype 3a and 3b Chinese Infections: Evolutionary Kinetics and Selective Force Analysis
Source: Viruses. 2022 Jul 11;14(7):1514. doi: 10.3390/v14071514 (PMC9324606; doi:10.3390/v14071514)

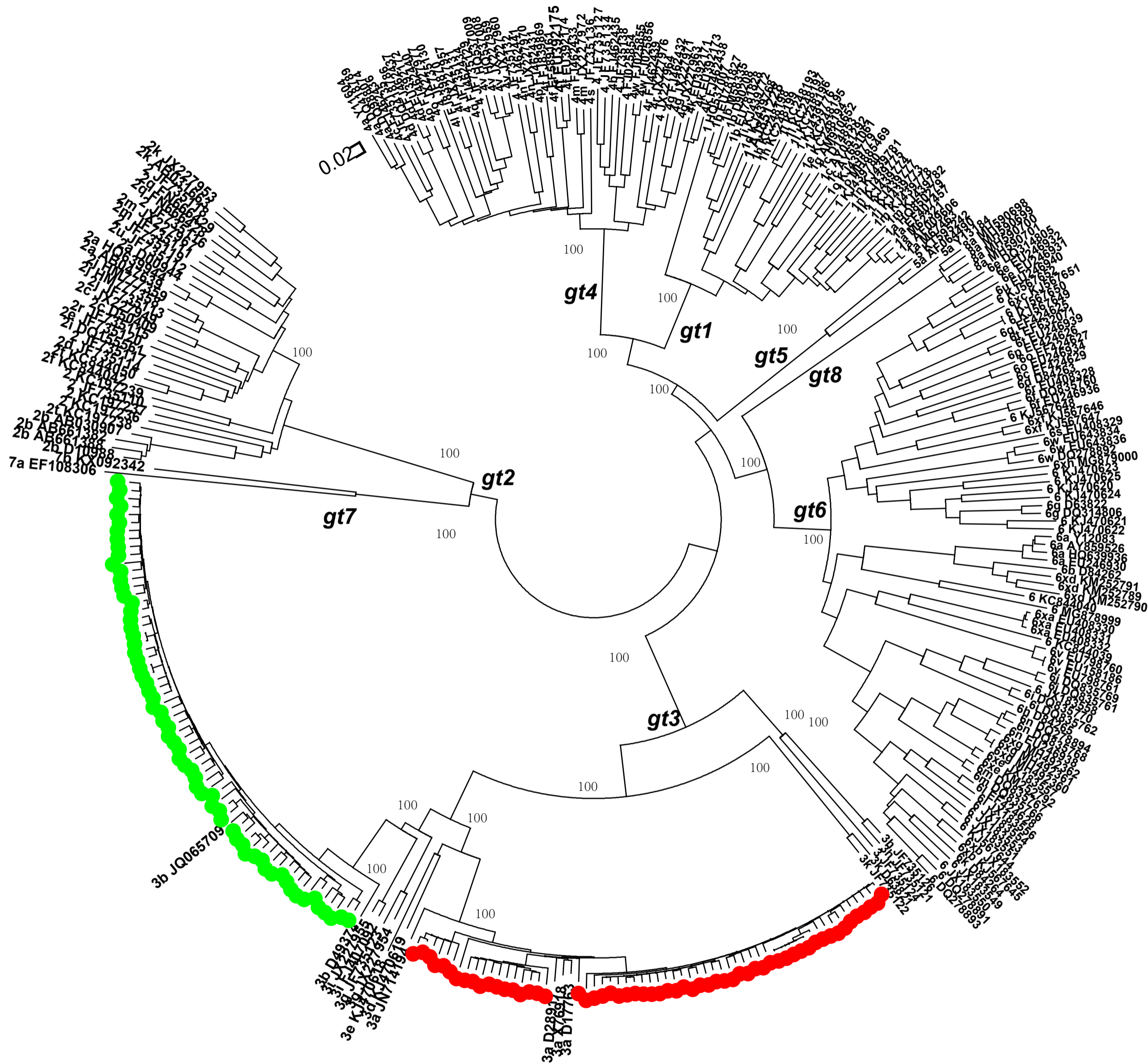

Supplement: Supplementary file 1 [file viruses-14-01514-s001.zip › viruses-1726694-Figure S2.pdf]
